# Supplementary material for: Characterization of Endothelial Cell Subclusters in Localized Scleroderma Skin with Single-Cell RNA Sequencing Identifies NOTCH Signaling Pathway
Source: Int J Mol Sci. 2024 Sep 28;25(19):10473. doi: 10.3390/ijms251910473 (PMC11477421; doi:10.3390/ijms251910473)
Supplement: Supplementary file 1 [file ijms-25-10473-s001.zip › Final Supplemental Legends.pdf]

The following supporting information can be downloaded at: www.mdpi.com/xxx/s1, Figure S1: Additional Endothelial Cell Object Data; Figure S2: Arterial EC as sender and All EC as Receiver NicheNet Analysis; Figure S3: Capillary EC as Sender and All Cells as Receiver NicheNet Analysis; Figure S4: Spatial analysis of JAG/NOTCH Pathway Genes; Figure S5: Proliferating EC as Sender and All EC as Receiver Nichenet Analysis; Figure S6: Important Gene Feature Plots; Figure S7: PVC and PCV DEG analysis; Table S1: Demographic Information; Table S2: All-cells Seurat Object Metadata; Table S3: Endothelial Cell Object Metadata.

Table S1: Demographic Information. Demographic information of our 44 samples dataset which includes 27 LS samples and 17 healthy samples. Information is broken into four subgroups: LS adults (n = 13), LS pediatrics (n = 14), healthy adults (n = 11), and healthy pediatrics (n = 6).

Table S2: All-cells Seurat Object Metadata. A summary of the metadata of our Seurat object containing all cells (108239 total cells). Included are counts of each cell type by health, onset, sex and ethnicity.

Table S3: Endothelial Cell Object Metadata. A summary of the metadata of our endothelial cell Seurat object that includes 16766 cells. Included are counts of each endothelial cell type by health, onset, sex and ethnicity.

Figure S1: Additional Endothelial Cell Object Data. **(A)** Sub-clustering output used to define the subclusters of endothelial cell subset which includes 13 subclusters at a resolution of 0.5. **(B)** Top DEGs when ran on endothelial cells versus all other cell types. **(C)** Distribution of healthy vs LS cells in endothelial cell subset. **(D)** Proportion of each endothelial cell subtype within LS cells and healthy cells.

Figure S2: Arterial EC as sender and All EC as Receiver NicheNet Analysis. This figure shows the NicheNet output for the arterial as sender and all cells as receiver analysis. **(A)** Top predicted ligands displayed that resulted from arterial as sender and all cells as receiver along with likely interaction with other cells in total dataset**.** **(B)** Heatmap demonstrating the target genes the ligands interact with. **(C)** Ligand and receptor interaction potential matrix for the top 20 predicted ligands. **(D)** CXCL signaling network, predicted to be significant within this analysis. The highest communication probabilities between sender and receiver cell types includes pericytes and venous ECs, pericytes and PVCs, pericytes and PCVs, PVCs and venous ECs, PVCs and PVCs, and PVCs and PCVs respectively.

Figure S3: Capillary EC as Sender and All Cells as Receiver NicheNet Analysis. **(A)** Top 20 predicted ligands when sent from capillary ECs and the cell types they communicate with. **(B)** Matrix displaying the regulatory potential of the target genes relative to the top 20

predicted ligands sent from capillary ECs. **(C)** Matrix of the top 20 predicted capillary EC ligands and receptors they are likely to interact with in the dataset. **(D)** CCL signaling network includes relevant sender and receiver cell types, with the top interactions being between pericytes and venous, PVCs and PCVs, and PVCs and venous ECs, PVCs and PCVs respectively.

Figure S4: Spatial analysis of JAG/NOTCH Pathway Genes. Figures **(S4A-E)** displays an affected skin biopsy from the right thigh of an LS patient that underwent spatial transcriptomics. The expression scale is in 0-5/6 Log base 2. **(A)** H&E stain on the sample, with an area of inflammatory infiltrate circled. Figures **(S4B and S4C)** respectively display where JAG1 and JAG2 localize spatially. **(D and E)** respectively display where NOTCH1 and NOTCH2 localize spatially. NOTCH4 was not found in the spatial software search, so NOTCH2 was used instead.

Figure S5: Proliferating EC as Sender and All EC as Receiver Nichenet Analysis. **(A)** Top 20 predicted ligands when sent from proliferating ECs and cell types they communicate with. **(B)** Matrix displaying the regulatory potential of the target genes relative to the top 20 predicted ligands sent from proliferating ECs. Highlighted in blue text includes ligands IL6, APOE, ICAM1, HMGB1, LGALS3 and IL33. **(C)** Matrix of the top 20 predicted proliferating EC ligands and what receptors they are likely to interact with in our dataset.

Figure S6: Important Gene Feature Plots. **(A)** Localization of IL33 on all cells object, identifying its location as only in the endothelial cell cluster. **(B)** Feature pots of IL33, IL6 and CCL21 split by health. IL33 spans through all endothelial cell clusters, IL6 localizes mostly in the PVCs and the PCVs. CCL21 localizes in the lymphatic endothelial cells. **(C)** is for reference and shows the labeled endothelial subset dimplot.

Figure S7: PVC and PCV DEG analysis. **(A)** Volcano plot of the top upregulated genes in the pre-venular capillary (PVC) subcluster. **(B)** GSEA overlap output of the top 200 pre-venular capillary (PVC) LS differentially expressed genes. **(C)** Volcano plot of the differentially expressed genes of the post-capillary venules subcluster **(D)** GSEA overlaps output from the top 200 post-capillary venule LS differentially expressed genes. **(E-F)** Top LS differentially expressed genes for the PVCs and PCVs respectively which includes SELE and XIST for both.
