# Supplementary material for: Characterization of Endothelial Cell Subclusters in Localized Scleroderma Skin with Single-Cell RNA Sequencing Identifies NOTCH Signaling Pathway
Source: Int J Mol Sci. 2024 Sep 28;25(19):10473. doi: 10.3390/ijms251910473 (PMC11477421; doi:10.3390/ijms251910473)
Supplement: Supplementary file 1 [file ijms-25-10473-s001.zip › Table S1.pdf]

Category	Category name	Sample ID	Chemistry	Sample Type	Bx Location	Onset	Age of onset	Disease Duration	Age at biopsy (yrs)	Gender	Race/Ethnicity	Active/ Inactive	Subtype	Antibody Status	LoSAI/ mLoSSI	LoSDI	PGA-A	PGA-D	PGA-S
1A	adult control	SC50	V2	Fresh	Forearm	Adult	N/A	N/A	64	M	Caucasian	N/A	Healthy	N/A	N/A	N/A	N/A	N/A	N/A
1A	adult control	SC68	V2	Fresh	Forearm	Adult	N/A	N/A	48	F	Caucasian	N/A	Healthy	N/A	N/A	N/A	N/A	N/A	N/A
1A	adult control	SC124	V2	Fresh	Forearm	Adult	N/A	N/A	54	M	Caucasian	N/A	Healthy	N/A	N/A	N/A	N/A	N/A	N/A
1A	adult control	SC125	V2	Fresh	Forearm	Adult	N/A	N/A	61	M	African American	N/A	Healthy	N/A	N/A	N/A	N/A	N/A	N/A
1A	adult control	SC1	V1	Fresh	Forearm	Adult	N/A	N/A	63	M	Caucasian	N/A	Healthy	N/A	N/A	N/A	N/A	N/A	N/A
1A	adult control	SC4	V1	Fresh	Forearm	Adult	N/A	N/A	63	M	Caucasian	N/A	Healthy	N/A	N/A	N/A	N/A	N/A	N/A
1A	adult control	SC18	V1	Fresh	Forearm	Adult	N/A	N/A	66	F	Caucasian	N/A	Healthy	N/A	N/A	N/A	N/A	N/A	N/A
1A	adult control	SC33	V1	Fresh	Forearm	Adult	N/A	N/A	62	F	Caucasian	N/A	Healthy	N/A	N/A	N/A	N/A	N/A	N/A
1A	ped control	SC407	V3	Cryostor	Breast	Peds	N/A	N/A	18	F	Caucasian	N/A	Healthy	N/A	N/A	N/A	N/A	N/A	N/A
1A	ped control	SC392	V3	Fresh	Forearm	Peds	N/A	N/A	23	M	Caucasian	N/A	Healthy	N/A	N/A	N/A	N/A	N/A	N/A
1A	ped control	SC386	V3	Fresh	Breast	Peds	N/A	N/A	21	F	Caucasian	N/A	Healthy	N/A	N/A	N/A	N/A	N/A	N/A
1B	ped control	SC32	V1	Cryostor	Forearm	Peds	N/A	N/A	23	F	Asian	N/A	Healthy	N/A	N/A	N/A	N/A	N/A	N/A
1B	ped control	SC296	V2	Cryostor	Scalp	Peds	N/A	N/A	5	M	Caucasian	N/A	Healthy	N/A	N/A	N/A	N/A	N/A	N/A
1B	ped control	SC297	V2	Cryostor	Midarm	Peds	N/A	N/A	14	F	Hispanic	N/A	Healthy	N/A	N/A	N/A	N/A	N/A	N/A
1B	ped control	HSK053	V2	Fresh	Breast	Peds	N/A	N/A	18	F	African American/ Caucasian	N/A	Healthy	N/A	N/A	N/A	N/A	N/A	N/A
1B	ped control	HSK054	V2	Fresh	Breast	Peds	N/A	N/A	17	F	African American	N/A	Healthy	N/A	N/A	N/A	N/A	N/A	N/A
1B	ped control	PHC003	V2	Cryostor	Midarm	Peds	N/A	N/A	13	F	Hispanic	N/A	Healthy	N/A	N/A	N/A	N/A	N/A	N/A
2A	adult LS	SC445	V3	Cryostor	Abdomen	Adult	27	12	27	F	African American	A	Linear	-	5	2	21	14	-
2A	adult LS	SC446	V3	Cryostor	Abdomen	Adult	65	96	65	M	Caucasian	A	Generalized	-	13	17	32	8	-
2A	adult LS	SC275	V2	Cryostor	Left flank	Adult	63	2	65	F	Caucasian	A	Generalized, Isomorphic morphea	unknown	5	32	10	23	-
2A	adult LS	SC222AC	V2	ffected Cryostor	Left abdomen	Adult	-	-	65	M	Caucasian	A/I	Generalized	unknown	14	38	18	18	-
2A	adult LS	SC260	V2	Fresh	Lower back	Adult	34	13	43	M	Caucasian	I	Circumscribed/ Plaque	unknown	0	5	0	18	12
2A	adult LS	HJ809	V2	Cryostor	Abdomen	Adult	64	1	65	F	Caucasian	A	Generalized	unknown	61	28	90	60	-
2A	adult LS	HJ867	V2	Cryostor	Right Arm	Adult	54	8	61	F	Caucasian	A	Generalized	unknown	13	39	27	30	-
2A	adult LS	SC408	V3	Cryostor	Abdomen	Adult	66	46	66	F	Caucasian	A	Generalized	-	5	26	4	17	-
2A	adult LS	SC409	V3	Cryostor	Right thigh	Adult	47	7	47	F	African American	A	Indeterminate	-	12	17	19	12	-
2A	adult LS	SC389	V3	Cryostor	Abdomen	Adult	71	131	71	F	Hispanic	A	Linear	-	5	13	8	12	-
2A	adult LS	SC266	V2	Cryostor	Abdomen	Adult	24	13	37	M	Caucasian	A	Linear	unknown	4	34	13	55	-
2A	adult LS	SC267	V2	Cryostor	Left abdomen	Adult	61	2	64	F	Caucasian	A	Generalized	unknown	9	14	23	12	-
2A	adult LS	SC272	V2	Cryostor	Left abdomen	Adult	14	9	43	F	Hispanic	A	Linear	unknown	10	10	23	15	-
2B	ped LS	SC126	V2	Fresh	Upper thigh	Peds	6.3	9.19	8	F	Caucasian	A	Linear	ANA negative, Histone negative, ssDNA negative	17	14	76	35	43
2B	ped LS	SC198	V2	Fresh	Midarm	Peds	13	14.69	14	F	Asian	I	Linear	unknown	0	8	0	18	19
2B	ped LS	SC246	V2	Fresh	Thigh	Peds	12.6	13.97	15	F	Asian	A	Linear	ANA positive (1:160 homogenous), Histone+ ssDNA+	13	12	62	37	47
2B	ped LS	SC259	V2	Fresh	Scalp/foreheac	Peds	7.4	15.85	16	M	Caucasian	A	Linear Face	6/2019: Histone negative, ssDNA negative	4	4	29	41	45
2B	ped LS	SC144	V2	Fresh	Lower back	Peds	16.6	20.56	20	M	Caucasian	I	Circumscribed	not done at this visit	7	14	0	52	53
2B	ped LS	SC300	V2	Fresh	Scalp/foreheac	Peds	4.8	17.16	20	M	Caucasian	A	Linear Face	not done at this visit	3	5	8	47	52
2B	ped LS	SC424	V3	Cryostor	calp and Temp	Peds	10	1	17	F	Caucasian	A	Linear Face	negative ANA	0	3	5	51	53
2B	ped LS	SC442	V3	Fresh	Left Calf	Peds	5	88	13	F	Caucasian	A	Linear	not done at this visit	1	10	33	36	38
2B	ped LS	SC443	V3	Fresh	Left buttocks	Peds	17	3	16	F	Caucasian	A	Circumscribed	Positive ANA (homogenous, negative dsDNA, negative RNP)	4	5	46	22	29
2B	ped LS	SC457	V3	Fresh	Arm	Peds	4	12	5	F	Caucasian	A	Pansclerotic	not done at this visit	52	68	78	81	95
2B	ped LS	SC361	V2	Cryostor	Right Thigh	Peds	10	0.76	11	F	Caucasian	A	Linear	ANA negative, Histone negative	9	6	65	20	42
2B	ped LS	SC388	V3	Cryostor	Upper Back	Peds	10	69	16	F	Caucasian	I	Circumscribed	Histone negative	0	4	0	20	18
2B	ped LS	SC391	V3	Fresh	Right thigh	Peds	14	60	21	F	Caucasian	A	Linear	ANA positive (homogenous), histone negative	12	8	35	30	44
2B	ped LS	SC455	V3	Cryostor	Thigh	Peds	11	16	13	F	Caucasian	A	Linear	Positive Histone, negative PM1	2	5	14	42	40
