# Supplementary figures and images for: Characterization of Endothelial Cell Subclusters in Localized Scleroderma Skin with Single-Cell RNA Sequencing Identifies NOTCH Signaling Pathway
Source: Int J Mol Sci. 2024 Sep 28;25(19):10473. doi: 10.3390/ijms251910473 (PMC11477421; doi:10.3390/ijms251910473)

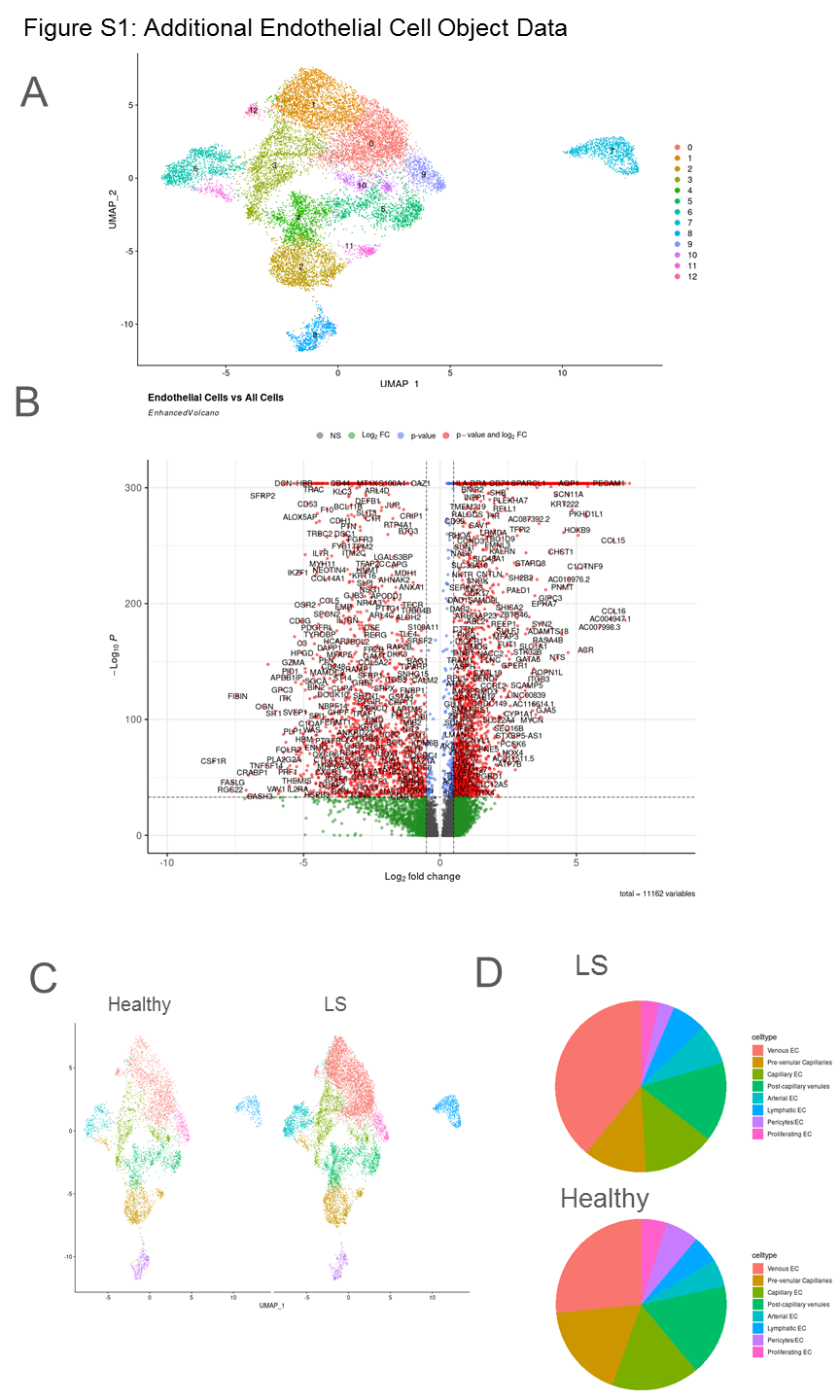

Supplement: Supplementary file 1 [file ijms-25-10473-s001.zip › Figure S1.PNG]

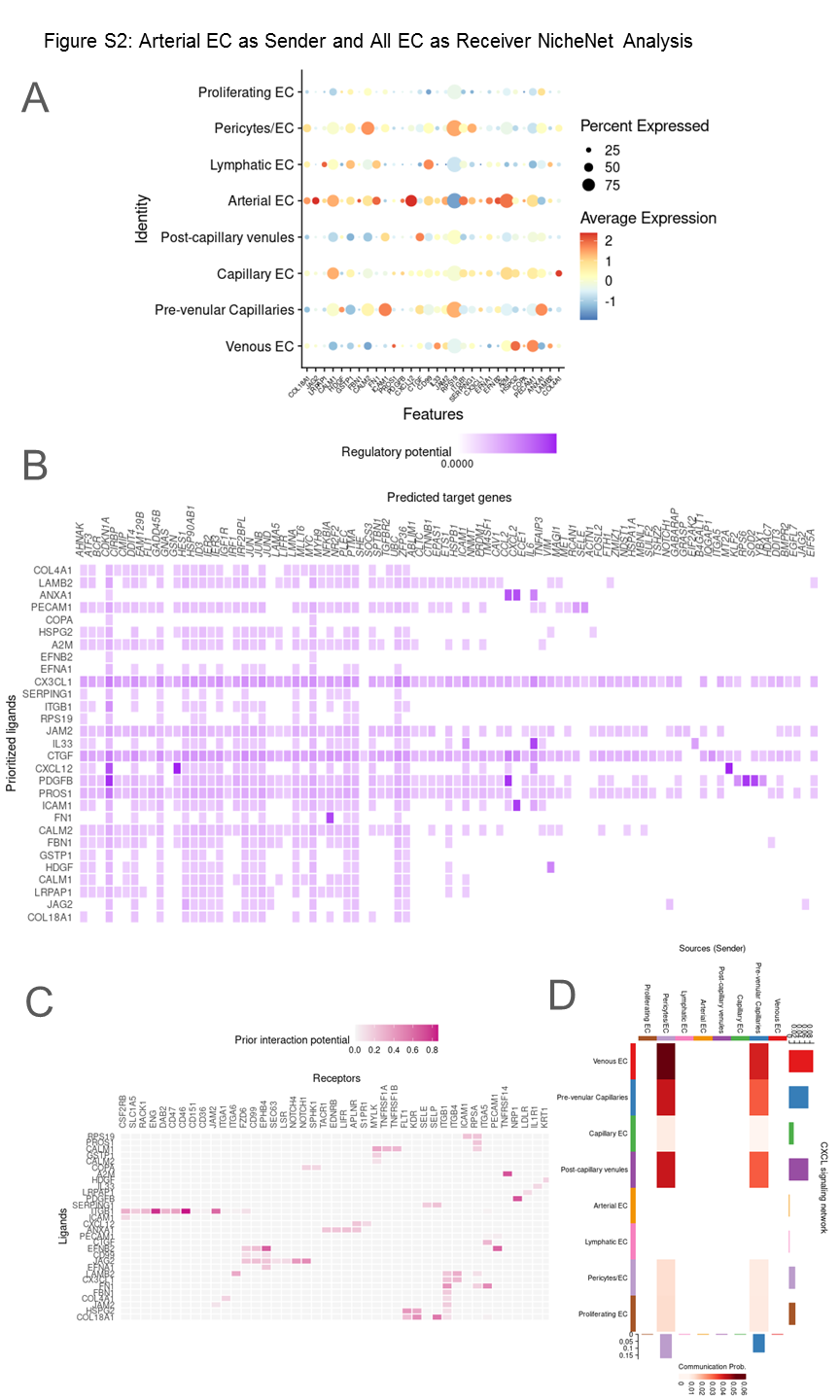

Supplement: Supplementary file 1 [file ijms-25-10473-s001.zip › Figure S2.PNG]

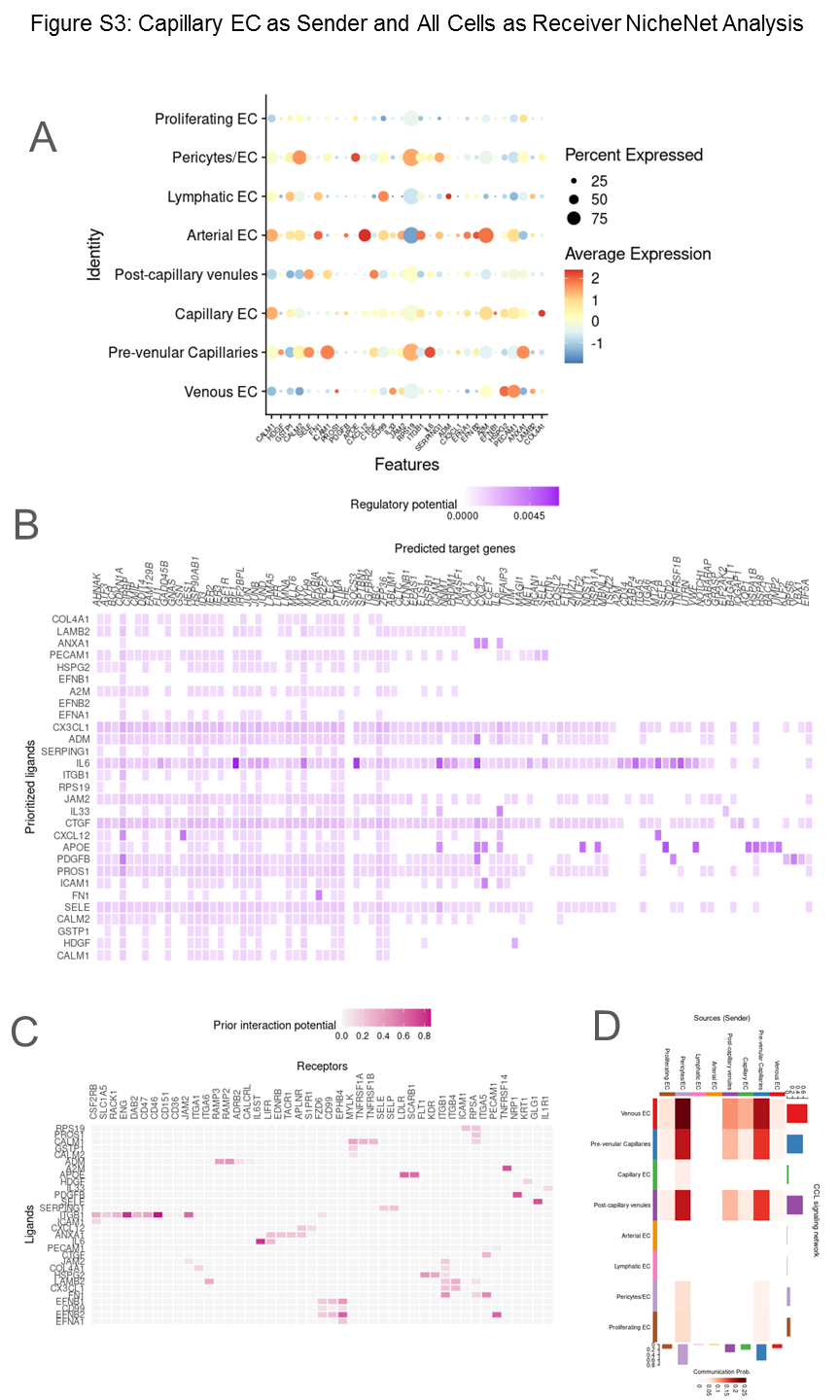

Supplement: Supplementary file 1 [file ijms-25-10473-s001.zip › Figure S3.PNG]

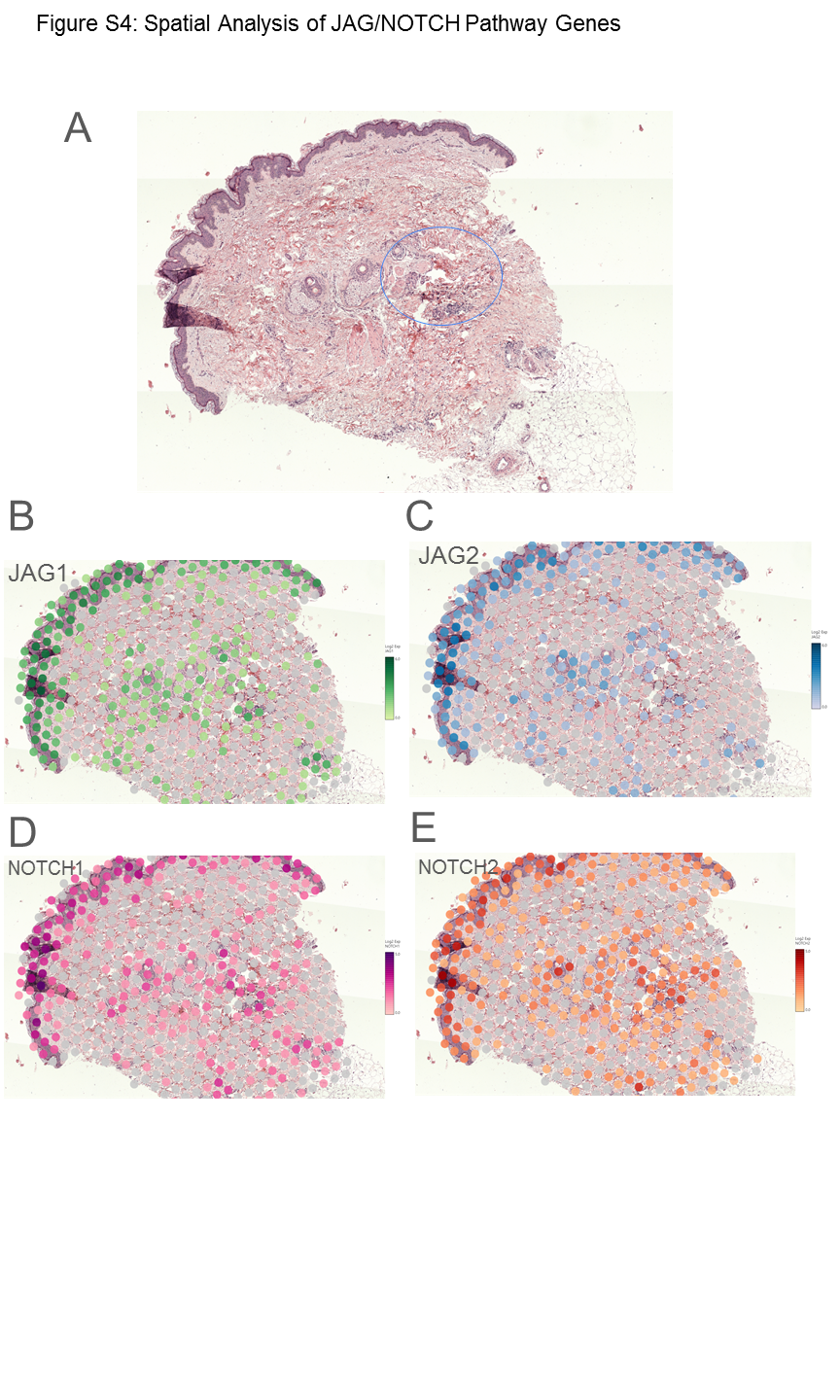

Supplement: Supplementary file 1 [file ijms-25-10473-s001.zip › Figure S4.PNG]

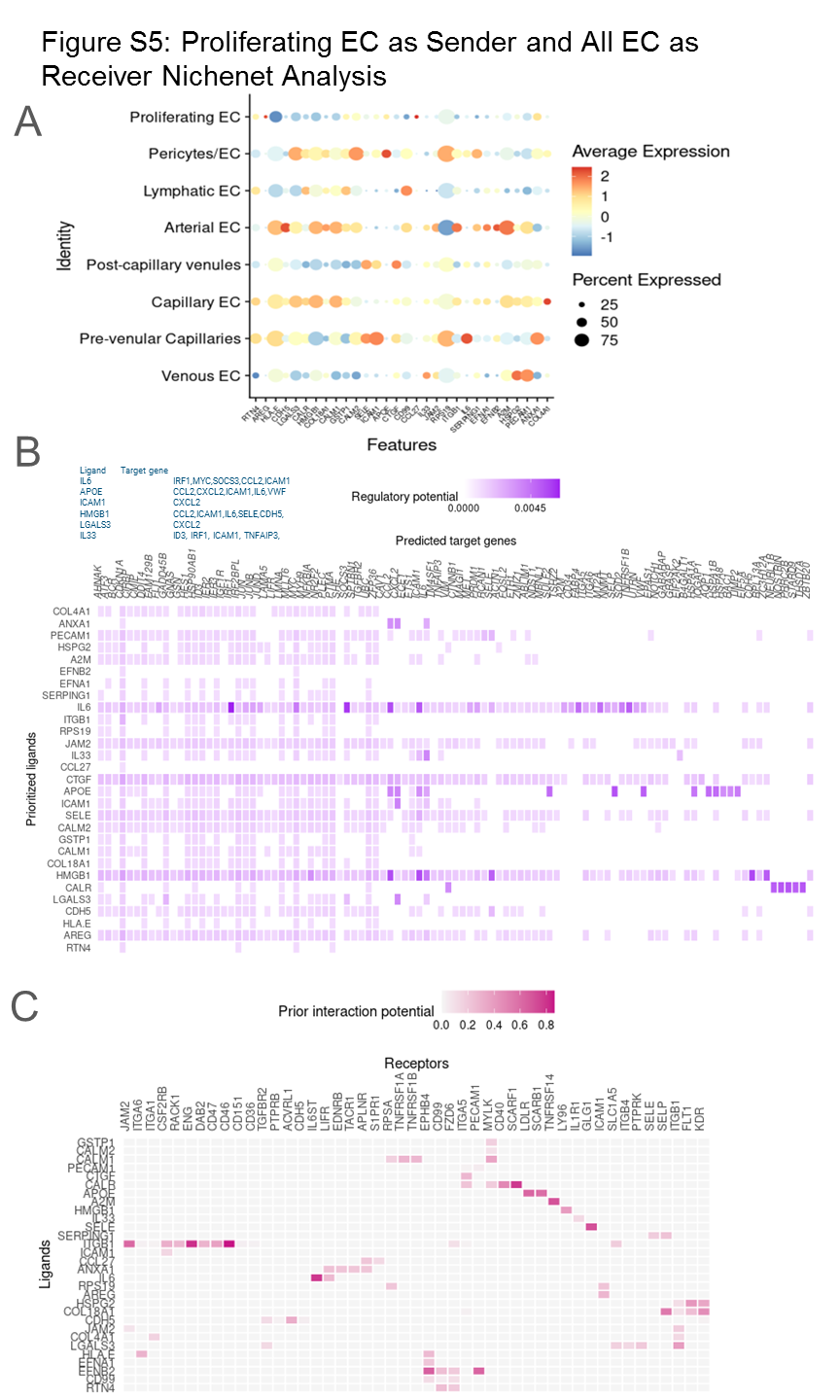

Supplement: Supplementary file 1 [file ijms-25-10473-s001.zip › Figure S5.PNG]

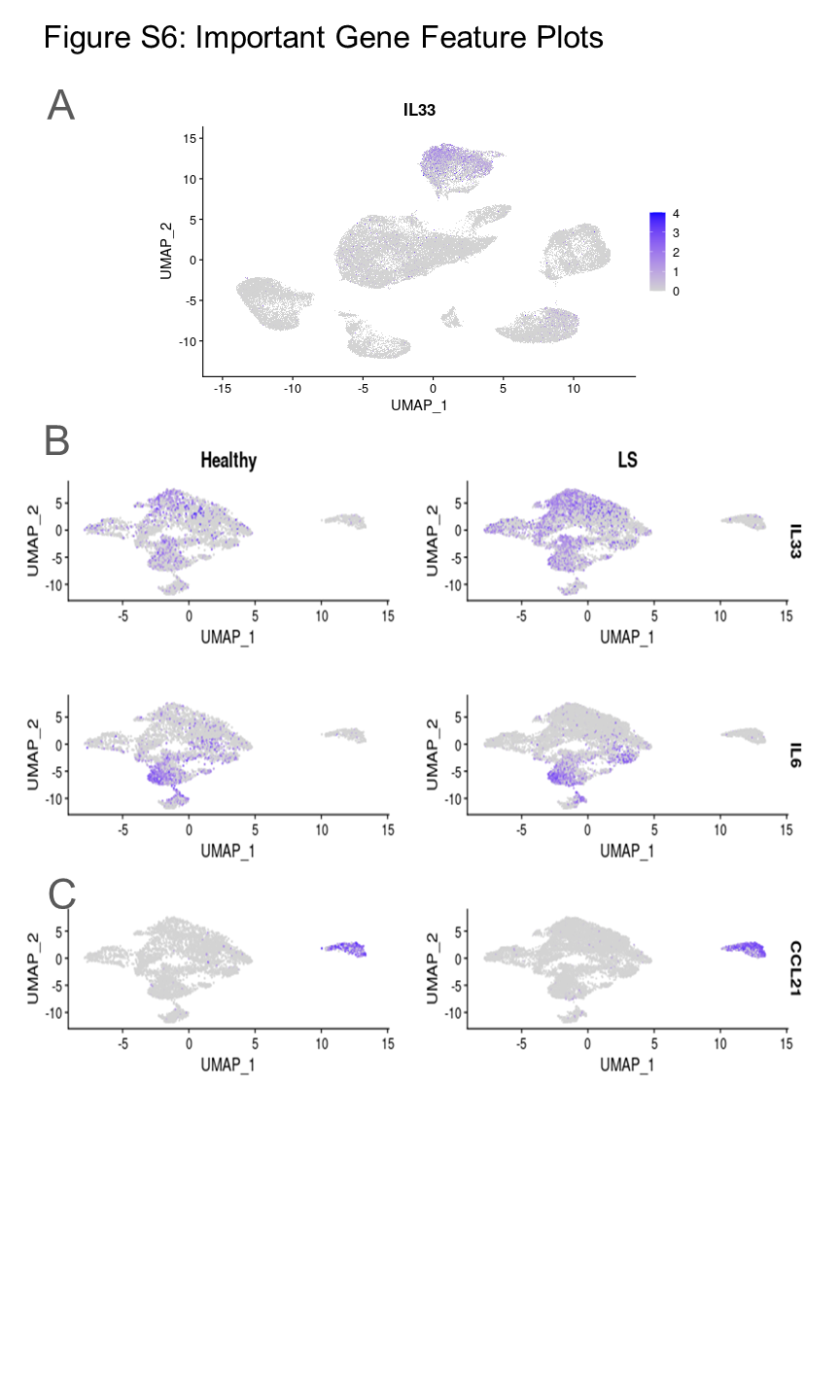

Supplement: Supplementary file 1 [file ijms-25-10473-s001.zip › Figure S6.PNG]

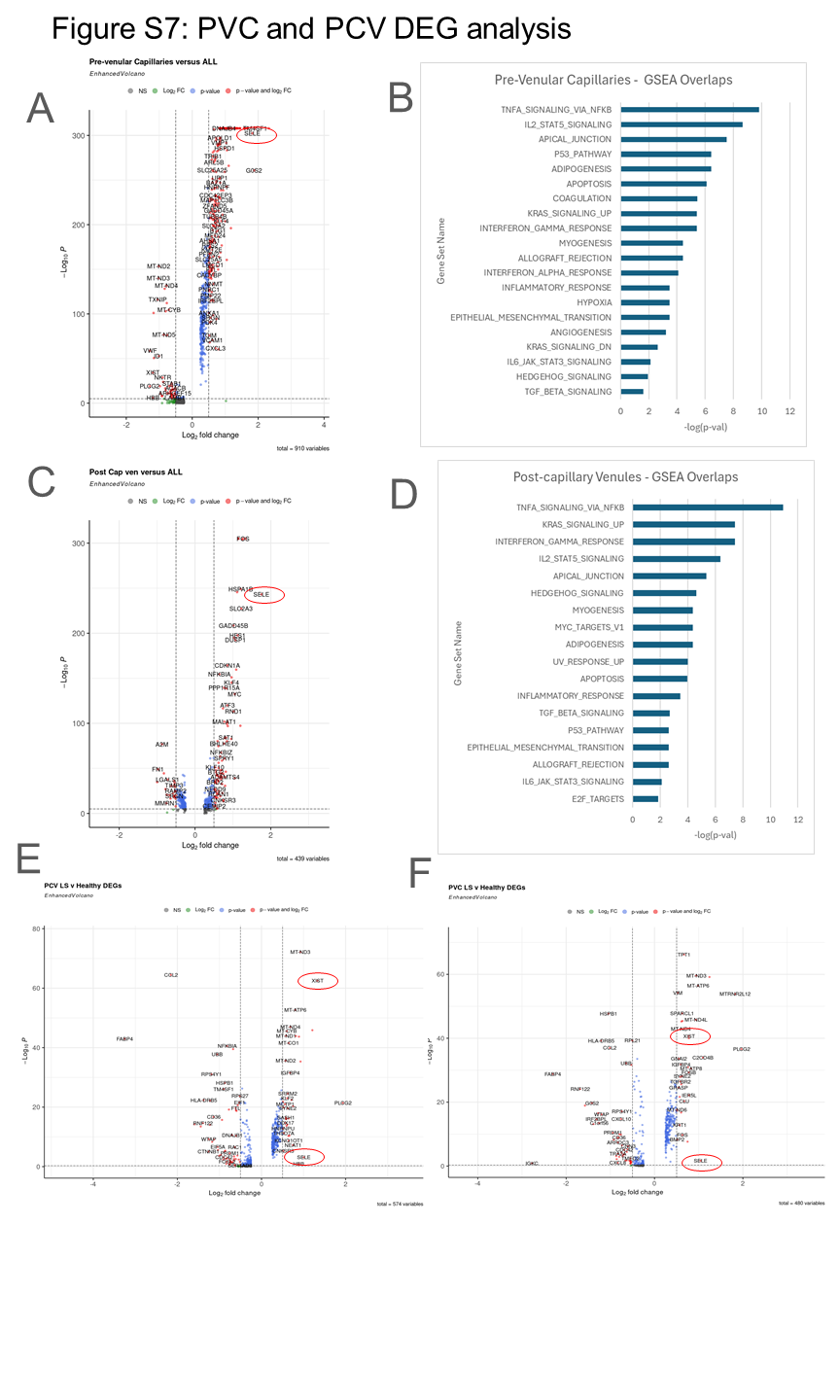

Supplement: Supplementary file 1 [file ijms-25-10473-s001.zip › Figure S7.PNG]

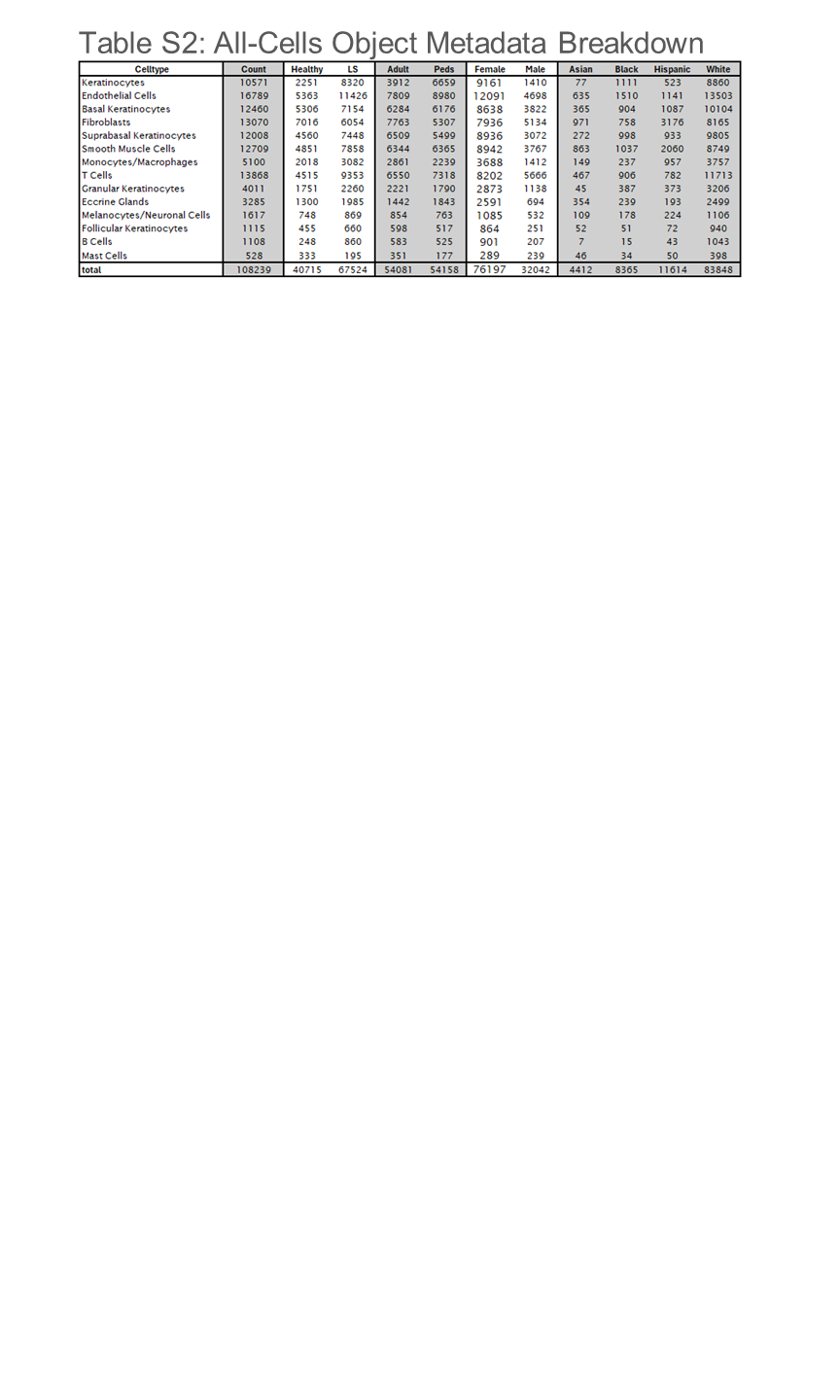

Supplement: Supplementary file 1 [file ijms-25-10473-s001.zip › Table S2.PNG]

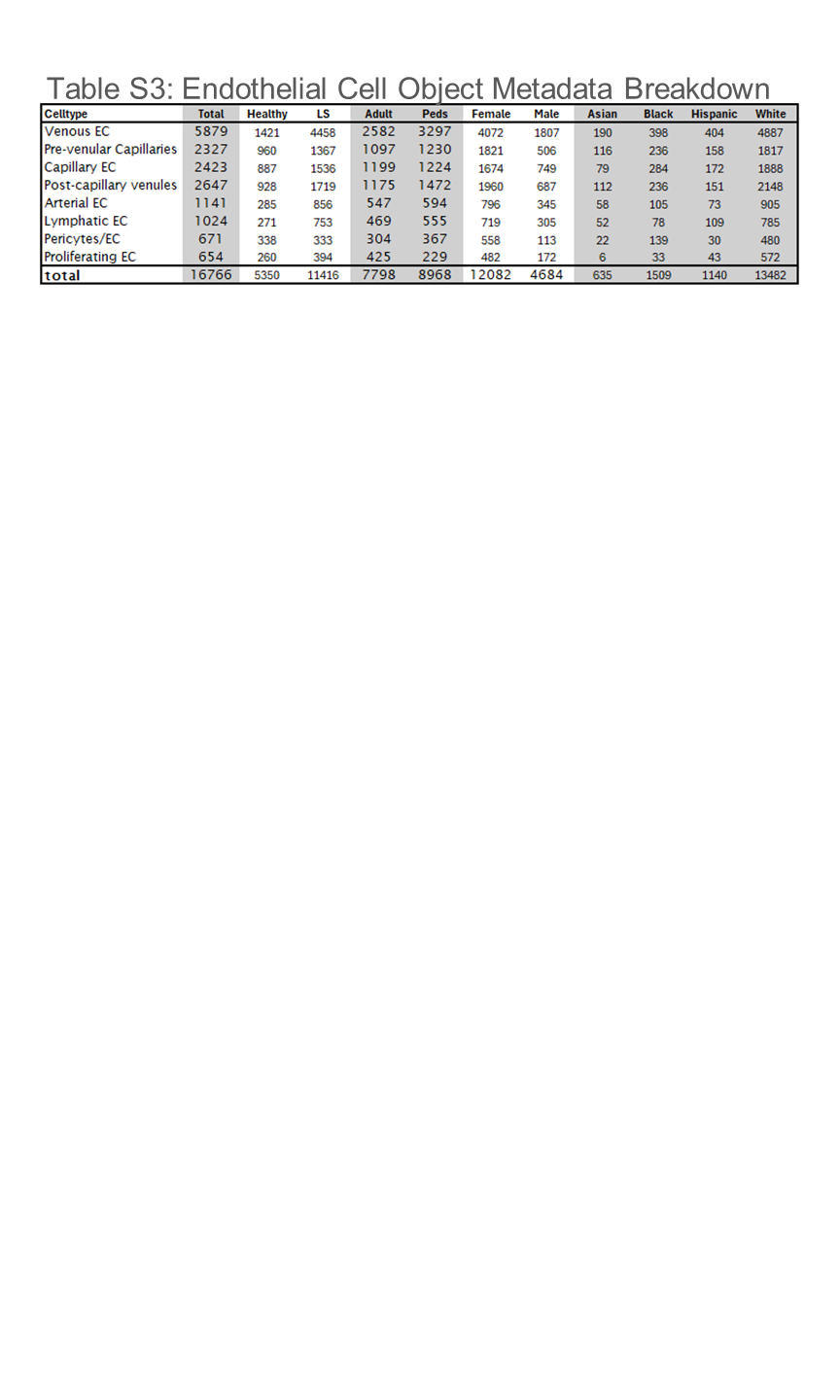

Supplement: Supplementary file 1 [file ijms-25-10473-s001.zip › Table S3.PNG]
